# How Pulmonary Embolism Burden Shapes Left Ventricular Strain and Recovery After Thrombectomy

**DOI:** 10.1016/j.jacadv.2026.103326

**Published:** 2026-09-22

**Authors:** Ziad Affas, Dhruva Govil, Johnathan Stephan, Vraj Bhatt, Bischan Hassunizadeh, Marcel Zughaib

**Affiliations:** a Department of Cardiology, Henry Ford Providence Southfield Hospital, Southfield, Michigan, USA; b Department of Internal Medicine, Henry Ford Providence Southfield Hospital, Southfield, Michigan, USA; c Baroda Medical College, Gurjarat, India

**Keywords:** left ventricular strain, pulmonary embolism, thrombectomy

**Figure:**
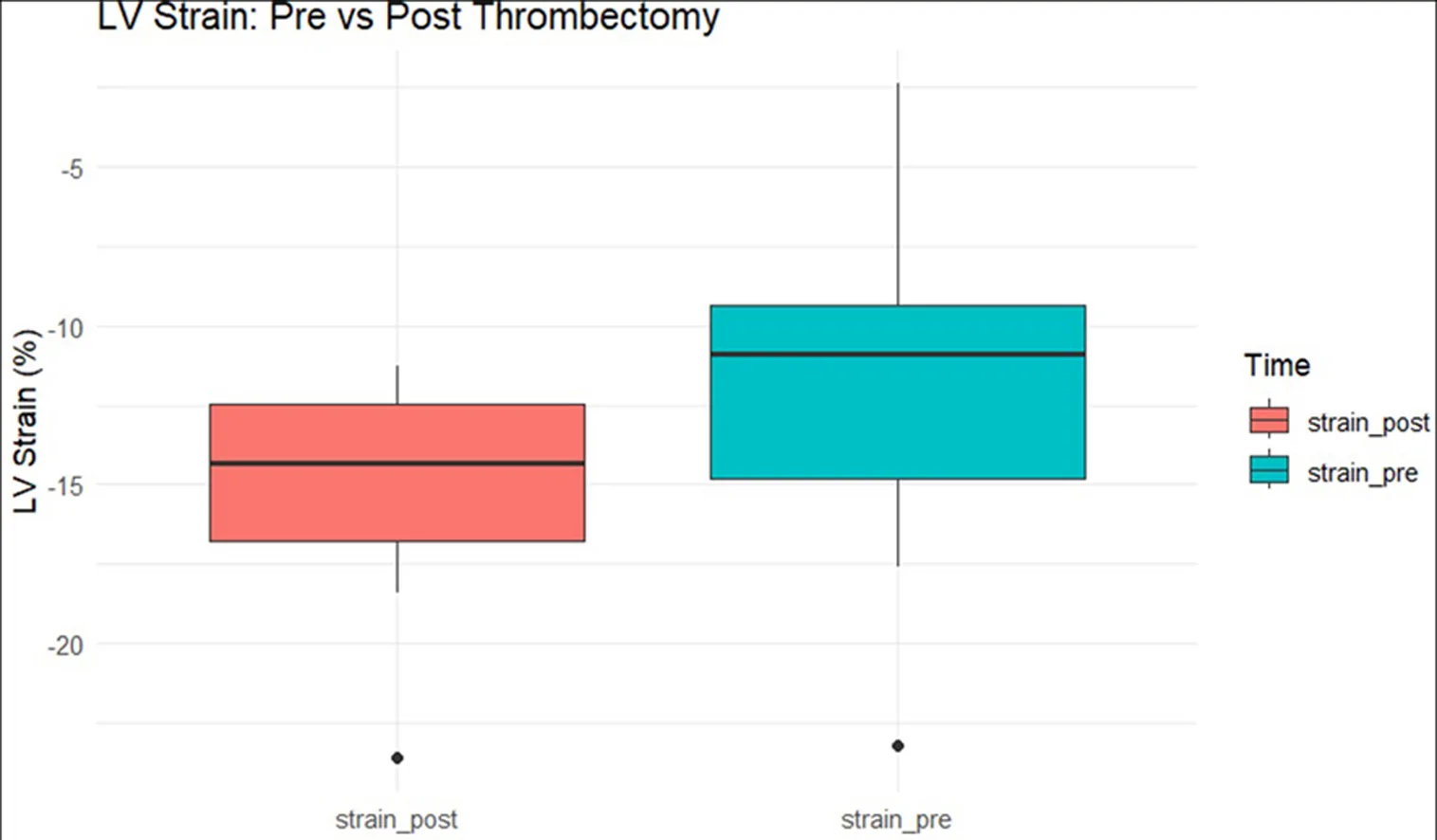

**What is the clinical question being addressed?**

Does left ventricular strain improve after mechanical thrombectomy in acute pulmonary embolism?

**What is the main finding?**

Left ventricular strain significantly improved following thrombectomy, with no observed linear association between strain improvement and pulmonary artery systolic pressure reduction.

Pulmonary embolism (PE) remains one of the leading causes of cardiovascular morbidity and mortality. Outcomes are largely determined by clot burden, right ventricular (RV) dysfunction, and hemodynamic compromise.^1^ Although RV impairment has been extensively studied, left ventricular (LV) strain in acute PE is increasingly recognized and thought to result from reduced preload and interventricular dependence.^2^^,^^3^ Global longitudinal strain (GLS), derived from speckle tracking echocardiography, is a sensitive marker of subclinical myocardial dysfunction and may detect abnormalities not captured by ejection fraction.^4^

The FLASH and FLAME trials have shown that mechanical thrombectomy has emerged as an important therapy for intermediate- and high-risk PE, demonstrating rapid hemodynamic improvement and reduction in RV strain.^4^^,^^5^ However, existing studies have paid little attention to LV strain or its trajectory following intervention. This gap is particularly relevant in the current era of expanding the use of catheter-based therapies for PE, where procedural success is typically defined by angiographic and RV parameters. A more comprehensive assessment that includes LV mechanics may better reflect global cardiac recovery and could refine postprocedural evaluation strategies.

This retrospective observational study included patients diagnosed with acute PE who underwent mechanical thrombectomy at Henry Ford Providence Hospital between 2017 and 2024. Patients with available prethrombectomy and post-thrombectomy transthoracic echocardiograms (TTE) suitable for strain analysis were included. Preprocedural TTEs were performed within 24 hours before thrombectomy, and all postprocedural TTEs were obtained within 3 days after thrombectomy. This short interval reduced, but did not eliminate, confounding from changes in clinical status or therapy.

The primary endpoint was the change in LV GLS following successful thrombectomy. Changes were calculated as postprocedure minus preprocedure values; more negative LV GLS values indicate improved myocardial deformation and systolic function. Secondary endpoints included changes in pulmonary artery systolic pressure (PASP) and LV ejection fraction (LVEF), as well as correlations between PE size, baseline LV strain, and post-thrombectomy improvement. Analyses were performed using the R system. Normality of paired difference scores was assessed using the Shapiro-Wilk test and Q-Q plots. Paired t-tests were used for approximately normal differences, with Wilcoxon signed rank tests as sensitivity analysis. Associations between changes in prethrombectomy and post-thrombectomy PASP and changes in LV GLS were assessed using Pearson correlation coefficients with 95% CIs. A 2-sided *P* value <0.05 was considered statistically significant. This Henry Ford Providence Hospital Institutional Review Board approved the study with waiver of informed consent.

Of the 71 catheterization laboratory activations for cardiology-performed mechanical thrombectomy, 5 were excluded due to non-PE indications (eg, right atrial thrombus), 7 lacked prethrombectomy echocardiograms, 19 lacked post-thrombectomy echocardiograms, 13 had insufficient image quality for strain analysis, and 10 had no echocardiographic data. After exclusions, 17 patients had successful thrombectomy and paired echocardiograms. Complete paired LV GLS measurements were available for 16 patients, who comprised the primary analysis cohort. LV GLS was measured using speckle-tracking echocardiography from apical views and averaged across segments. Successful thrombectomy was defined by angiographic thrombus removal, restoration of antegrade pulmonary flow, and visible clot burden reduction.

Among 16 patients with complete paired GLS measurements, the paired differences did not significantly depart from normality (Shapiro-Wilk W = 0.956; *P* = 0.596), and Q-Q plots showed no substantial deviation from normality. LV GLS improved from −12.2% ± 4.9% to −15.0% ± 3.3% (Δ = −2.84%; *P* = 0.01). The Wilcoxon signed rank sensitivity analysis yielded a consistent result (*P* = 0.008). PASP decreased from 41.0 ± 10.2 mm Hg to 27.8 ± 9.2 mm Hg (*P* = 0.01). LVEF increased from 57.2% ± 6.1% to 65.9% ± 6.4% (*P* = 0.001). Post-thrombectomy LV strain improved with reduced dispersion, consistent with paired t test findings (*P* = 0.01). Pearson correlation analysis demonstrated no significant linear association between changes in LV strain and PASP (r = 0.009; 95% CI: -0.474 to 0.487; *P* = 0.974) (Figure 1).

**Figure 1:**
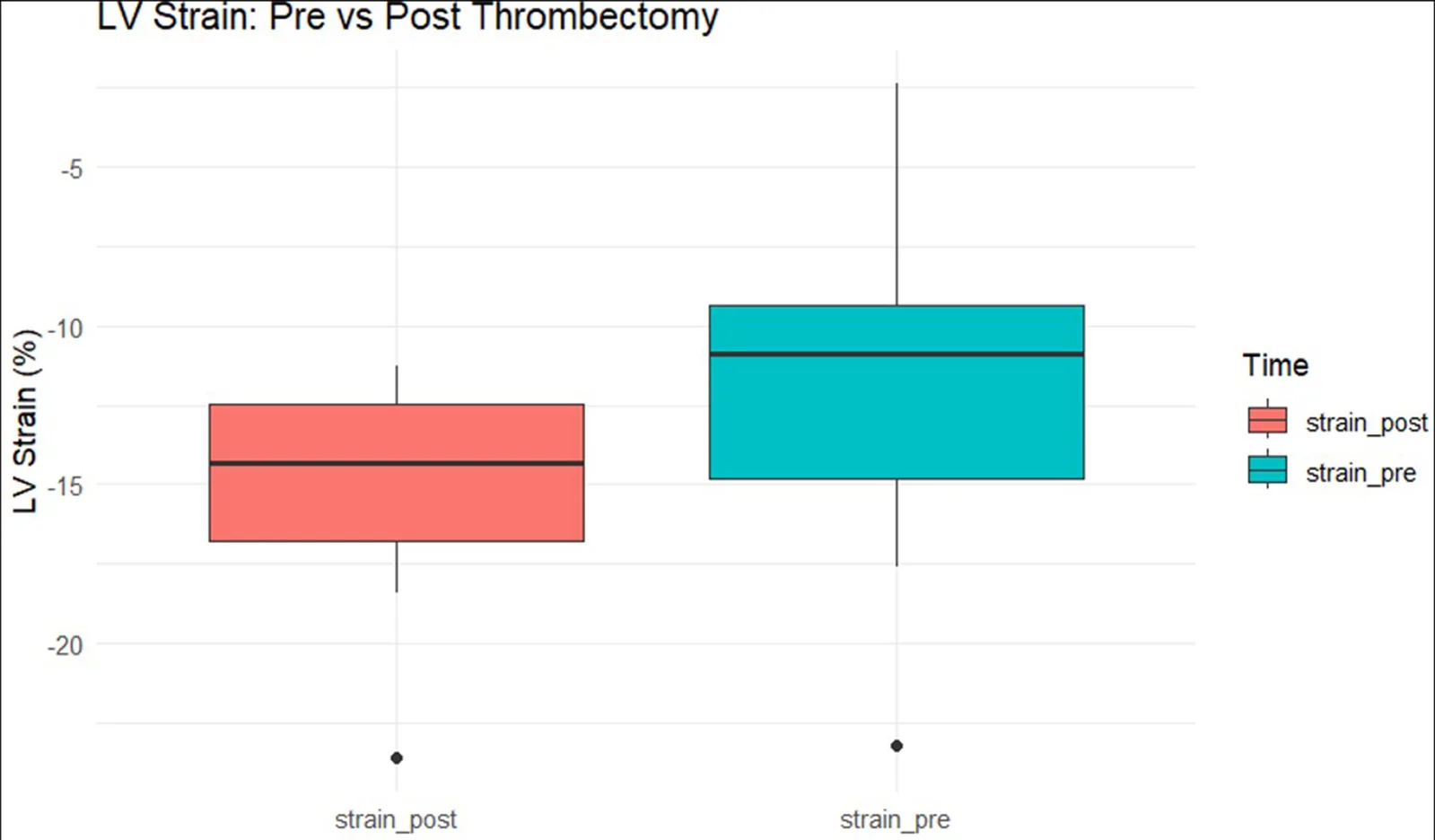
LV Strain: Pre- vs Post-Thrombectomy Box-and-whisker plots comparing LV global longitudinal strain measured before and after mechanical thrombectomy for acute pulmonary embolism in 16 patients with complete paired measurements. LV = left ventricular.

The magnitude of LV strain improvement exceeded the commonly cited threshold of approximately 2% considered clinically meaningful in prior strain studies, suggesting that the observed changes likely represent true physiologic recovery rather than measurement variability. In addition, although all patients demonstrated improvement in PASP, the degree of LV strain recovery varied across individuals, indicating heterogeneity in myocardial response despite uniform hemodynamic improvement.

This study demonstrated that LV strain improves following mechanical thrombectomy in acute PE. Thrombectomy improves forward flow across the pulmonary and aortic valves, reduces RV afterload, and restores LV filling, which likely contributes to improved myocardial deformation.

In addition to improved LV strain, the cohort demonstrated significant reductions in PASP and increases in LVEF, supporting a pattern of global cardiac recovery after thrombectomy. Concordant paired t-test and Wilcoxon signed rank results support the robustness of the observed LV GLS improvement despite the small sample size.

Prior studies, including FLARE and FLASH, have consistently documented improvements in RV/LV ratio and pulmonary pressures. However, LV strain has not been systematically evaluated in these cohorts.^5^ Our results add to the existing literature by demonstrating that LV strain also improves following intervention, providing an additional measure of cardiac recovery beyond traditional RV-focused metrics.

Importantly, no significant correlation was observed between changes in LV strain and PASP. Given the observational design and limited sample size, these findings should be interpreted cautiously. Although this study did not identify a significant association between these variables, the absence of an observed association suggests that LV strain may reflect aspects of myocardial recovery not fully captured by pressure-based measurements alone.

Although all patients demonstrated improvement in PASP, the degree of LV strain recovery varied across individuals. This heterogeneity suggests that myocardial recovery may not occur uniformly despite similar hemodynamic improvement. This variability may reflect the influence of factors such as duration of embolic burden, ventricular interdependence, or transient myocardial dysfunction. These findings align with the concept of ventricular interdependence, where acute RV pressure overload leads to septal shift and impaired LV filling. Relief of RV afterload allows restoration of LV preload and forward flow, although recovery of myocardial mechanics may lag hemodynamic improvement.

Clinically, these findings suggest that LV strain may provide incremental information beyond traditional RV metrics and may serve as a marker of biventricular recovery following thrombectomy. First, LV strain may be a useful addition to PE assessment, providing a more complete picture of cardiac involvement. Second, measuring LV strain before and after thrombectomy may help track recovery and identify patients who may benefit from closer follow-up.

This study is limited by its retrospective design, small sample size, and single-center experience. The highly selected study cohort included only patients with successful thrombectomy and adequate paired echocardiograms, which may limit generalizability and introduce selection bias. The study was underpowered to detect modest associations and may be affected by variability in image quality and strain software. Although all postprocedure echocardiograms were obtained within 3 days, residual confounding from changes in clinical status or therapy cannot be excluded.

In this observational cohort, LV strain improved following mechanical thrombectomy for acute PE. Although changes in LV strain were not significantly associated with changes in PASP, LV strain may serve as an adjunct marker of myocardial recovery following thrombectomy. Currently, these findings should be considered hypothesis-generating and warrant confirmation in larger prospective studies to better define the role of LV strain in assessing recovery following thrombectomy.

## Funding support and author disclosures

The authors have reported that they have no relationships relevant to the contents of this paper to disclose.
